# Cost analysis of the stroke volume variation guided perioperative hemodynamic optimization – an economic evaluation of the SVVOPT trial results

**DOI:** 10.1186/1471-2253-14-40

**Published:** 2014-05-22

**Authors:** Jan Benes, Jan Zatloukal, Alena Simanova, Ivan Chytra, Eduard Kasal

**Affiliations:** 1 Department of Anaesthesia and Intensive Care Medicine, Charles University Medical School and Teaching Hospital, Alej svobody 80, 304 60 Plzen, Czech Republic

**Keywords:** Hemodynamic optimization, Cost-effectiveness, Fluid optimization

## Abstract

**Background:**

Perioperative goal directed therapy (GDT) can substantially improve the outcomes of high risk surgical patients as shown by many clinical studies. However, the approach needs initial investment and can increase the already very high staff workload. These economic imperatives may be at least partly responsible for weak adherence to the GDT concept. A few models are available for the evaluation of GDT cost-effectiveness, but studies of real economic data based on a recent clinical trial are lacking. In order to address this we have performed a retrospective analysis of the data from the “Intraoperative fluid optimization using stroke volume variation in high risk surgical patients” trial (ISRCTN95085011).

**Methods:**

The health-care payers perspective was used in order to evaluate the perioperative hemodynamic optimization costs. Hospital invoices from all patients included in the trial were extracted. A direct comparison between the study (GDT, N = 60) and control (N = 60) groups was performed. A cost tree was constructed and major cost drivers evaluated.

**Results:**

The trial showed a significant improvement in clinical outcomes for GDT treated patients. The mean cost per patient were lower in the GDT group 2877 ± 2336€ vs. 3371 ± 3238€ in controls, but without reaching a statistical significance (p = 0.596). The mean cost of all items except for intraoperative monitoring and infusions were lower for GDT than control but due to the high variability they all failed to reach statistical significance. Those costs associated with clinical care (68 ± 177€ vs. 212 ± 593€; p = 0.023) and ward stay costs (213 ± 108€ vs. 349 ± 467€; p = 0.082) were the most important differences in favour of the GDT group.

**Conclusions:**

Intraoperative fluid optimization with the use of stroke volume variation and Vigileo/FloTrac system showed not only a substantial improvement of morbidity, but was associated with an economic benefit. The cost-savings observed in the overall costs of postoperative care trend to offset the investment needed to run the GDT strategy and intraoperative monitoring.

**Trial registration:**

ISRCTN95085011

## Background

Due to the recent economic situation in Europe financial resources in healthcare are becoming increasingly limited. Under these circumstances cost-effectiveness, or preferably cost savings, from new therapeutic approaches may be vital for their adoption, especially, if these new treatments are accompanied by higher initial acquisition or maintenance costs. Hemodynamic optimization and goal directed therapy (GDT) of high risk surgical patients improves postoperative outcomes by decreasing the number of complications and hospital length of stay as showed by many clinical trials and meta-analyses [1-3]. Additionally, according to pooled data from recently published meta-analysis an impact on postoperative mortality may be observed in the groups with high control-group mortality [4]. The reality though is that overall adoption of perioperative GDT is very low among European and American anaesthesiologists [5] and a lack of data on an economic benefit may be an important reason why. For example, if a comprehensive investment (financial and practice orientated) is needed, hospital administrators and insurance companies may not be willing to cover these expenses.

There are limited economic data regarding perioperative GDT published so far. In 1997 an economic evaluation of a study by Boyd [6] was performed by Guest [7] showing a decreased economic burden using pulmonary artery catheter for preoperative hemodynamic optimization. Later, similar results were published by Fenwick [8] based on the Wilson optimization trial [9]. Both of the original clinical studies used pulmonary artery catheter for guiding fluid optimization and in one [9] the patients were admitted to the hospital ICU the day before surgery in order to perform preoperative tuning-up. Nowadays, neither the device nor the preoperative GDT are relevant to contemporary practice. The development of less invasive devices has enabled the use of routine GDT in an intermediate to high risk population and studies performed intraoperatively and also in the early postoperative period have shown comparable results to preoperative treatment. All of these issues are highly relevant for potential economic decision making.

More relevant are two recent studies both using an economic modelling approach based on meta-analysis of clinical trials. Mowatt et al. [10] performed an economic evaluation of oesophageal Doppler use where for both the worst and best scenarios the use of this technology was deemed cost-effective. Another cost-effectiveness evaluation was performed by a Swedish group [11] where data from a mix of different devices and GDT strategies were used as model inputs applied to the clinical results of elderly patients with hip-fracture. These results also stressed the potential of perioperative GDT to be not only cost-effective but also cost saving with substantial clinical benefits. Nevertheless, despite these publications an evaluation based on real economic data coming from a study using less invasive device in high risk patients is lacking. To address this we decided to perform a retrospective, health care payers perspective, economic evaluation of the Intraoperative fluid optimisation using stroke volume variation in high risk surgical patients (SVVOPT) trial [12]. Our hypothesis was that the observed improvement in clinical outcomes with GDT would result in lower costs compared with the control arm.

The SVVOPT study used a novel, less invasive Vigileo/FloTrac system for the intraoperative fluid and hemodynamic optimization of high risk surgical patients. In total 120 patients were equally and randomly distributed into intervention (GDT, N = 60) and control group (Control, N = 60). The goal of fluid intervention in the GDT group was stroke volume variation (SVV) lower than 10% and cardiac index (CI) higher than 2.5 l/min/m^2^. Infusions of colloids and dobutamine were used in order to reach these goals. Considerable outcome benefit regarding number (34 vs. 78; p = 0.007) and rate (18 vs. 35 patients; p = 0.003) of postoperative complications was observed in the GDT group using an intention-to-treat approach. Additionally, in the per protocol analysis of patients with performed optimization the hospital length of stay was significantly shorter. There was no difference in number of deaths, but the study was not designed to be powered to prove a mortality benefit.

## Methods

The original SVVOPT study ran from July 2007 until May 2009 and enrolled 120 patients equally and randomly distributed into intervention and control groups. Written informed consent and approval of the local ethics committee of the Charles University Hospital in Plzen, Czech Republic was provided for the study. All economic data were routinely collected in the form of individual invoices issued for the healthcare payer at discharge. In order to avoid bias where possible due to the retrospective design, two of the authors here (JZ and AS) who were not part of the original trial and unaware of the patient treatment group allocation performed the extraction of the economic data. In order to evaluate the major drivers the costs were separated into 10 categories: anaesthesia, monitoring, infusion and blood product associated costs, further postoperative ward and ICU stay, clinical examination and procedures, laboratory diagnostics, antimicrobial agents, radio-diagnostics and others. In order to try and avoid large confounding factors, the costs of the surgical procedure, postoperative analgesia and preoperative stay were excluded from the final sum. However, in order to fully account the cost of GDT the monitoring relevant costs (Vigileo device and 60 FloTrac monitoring sets) were captured. These payments were not reimbursed by the payer, but are relevant for the future decision. As the study was performed in the Czech Republic some specifics of healthcare reimbursement should be mentioned:

• All clinical investigations are evaluated with a number of points, these points are fully reimbursed by the insurance company according to the actual course (this changed once during the study period and was increased accordingly).

• Some materials used (central venous catheters, parenteral nutrition, all antimicrobial agents, blood products, radio-contrast dye and other) are also reimbursed.

• Other materials (peripheral or arterial catheters, urinary catheters, usual medication, etc.) are reimbursed in lump sum payments and are billed in a *per diem* fee according to patient category of care.

• Finally, some medical services are neither charged to the patient nor the payer. From these only the costs of study specific material were included in the study: Vigileo machine – 6100 €, FloTrac monitoring sets - 175 € each, and intravenous infusions.

**Figure 1:**
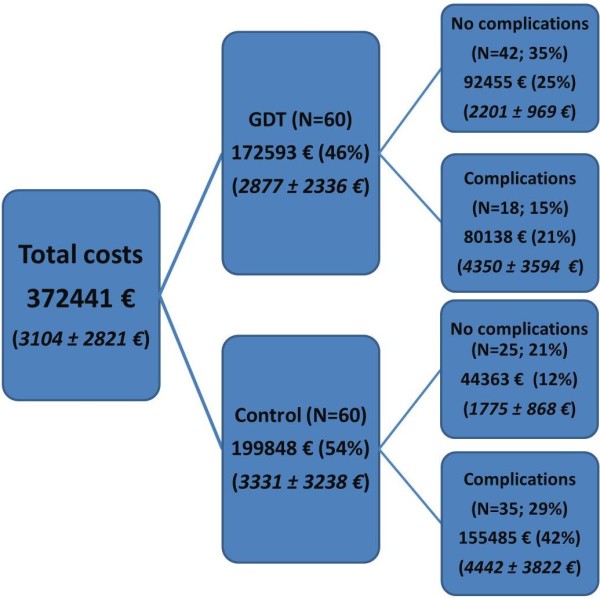
Cost tree for the two treatment arms of the SVVOPT study.

All sums were obtained in Czech Crowns (CZK) and converted to Euros (€) according to a mean exchange rate for the study period (25 CZK per 1 €). Any fluctuations in this rate according to data obtained from the Czech National Bank were very small making the potential bias irrelevant. No consideration of inflation was included in the calculations due to the relatively short study period.To assess the economic benefit of the intervention the following analyses were performed: Firstly, the total costs of both study groups and subcategories of patients with and without complications were analyzed. A cost tree (Figure 1) was constructed for these variables: treatment allocation as first branch and complication occurrence (as the major clinical outcome) as the second branch. For each branch a number of patients, total costs and their proportions were calculated. It is difficult to assess the cost-effectiveness ratio for GDT versus control in this retrospective study as it was underpowered to demonstrate a mortality benefit and although there was an obvious morbidity benefit (i.e. fewer complications for GDT) no patient related endpoints such as quality of life were prospectively collected. Therefore rather than complicate the analysis unnecessarily the results here are presented only in the form of costs. A discussion on the implications of this are included at the end of this report.

In addition to the overall cost analysis an analysis of specific reimbursement groups was performed in order to identify the major cost drivers and their importance to the total costs according to the treatment allocation. The costs are reported as both mean (standard deviation) and median (interquartile range) as proposed by Briggs [13] as means are less prone to reduce the impact of extreme values and hence enable better description of costs across a patients sample. However, nonparametric statistic tests (Mann–Whitney test, Kruskal-Wallis test) were used to assess the difference between study groups accordingly. All calculations were performed using the MedCalc software ver. 12.4.0.0. (MedCalc Software, Acacialaan 22, B-8400 Ostend, Belgium).

Finally, although this study is performed directly from patient-specific clinical and billing records, for most readers the costs of different complications may be of importance especially for the international comparison. Therefore, we have identified patients having specific complications in order to evaluate the induced additional costs. Patients were grouped according to the nature of their complications (i.e. none, solely infectious, solely non-infectious, a mixture). In addition, patients having a single complication were evaluated for additional costs.

## Results

Major clinical results of the study were already mentioned and are given in detail elsewhere [12]. Most relevant for this economic analysis are the following: the intervention reduced the number of complications (34 vs. 78 complications; p = 0.007) and their rate (18 vs. 35 patients in the GDT and Control group respectively; p = 0.003). The overall hospital length of stay (calculated as the sum of all stays per group) was 627 vs. 925 days for the GDT and Control group respectively. In the per protocol analysis, the median hospital stay was 9 days (8 to 12) in the GDT group compared to 10 days (8 to 19) in controls (p = 0.042).The mean total costs per patient in the intervention group were 2877 ± 2336 € (mean ± SD) whereas in the control group they were 3331 ± 3238 €. The overall cost was 27,255 € higher in the Control group and mean cost per patient was 454 € in favour of GDT, with large variability. As described in the cost tree (Figure 1) the mean cost of those without complications was slightly higher in the GDT group. Unsurprisingly, the study monitoring costs contributed the majority (70%) of this difference. Also, the overall mean costs of patients with complications did not differ substantially between groups. In total, patients with complications though minor in number (N = 53; 44%) were responsible for the majority of costs (235,623 €, 63%). The incidence of complications was the major driving parameter for increased total and mean costs in the Control group.

An overview of the major health care cost categories is presented in Table 1. The average costs of care tended to be lower for GDT versus Control (2877 ± 2336 € vs. 3331 ± 3238 €; p = 0.596) although not statistically significant. In fact all costs except those for intraoperative monitoring and infusions were lower for GDT than control but due to the high variability most failed to reach statistical significance. However, the study was not designed to power this comparison and a post-hoc analysis estimates a sample size of at least 200 patients in each arm to reach statistical significance. Thus a tendency for lower costs of postoperative care was observed (1891 ± 2170 € vs. 2501 ± 3152 €; p = 0.177) and clinical care related costs (honoraria, examinations and procedures) were significantly decreased in the GDT group (68 ± 177 € vs. 212 ± 593 €; p = 0.023). For other parameters, i.e. ward stay costs (213 ± 108 € vs. 349 ± 467 €; p = 0.082) and infection associated costs (99 ± 151 € vs. 236 ± 535 €; p = 0.365) only a trend in favour of the GDT group was observed. As monitoring related costs (Vigileo machine and monitoring FloTrac kits) were included in the calculation the costs of intraoperative care were bound to be higher in the GDT group (986 ± 351 € vs. 830 ± 469 €; p = 0.001) but in general, mean intraoperative costs were higher 156 ± 351 € (p = 0.001) and postoperative costs lower 609 ± 3152 € (p = 0.177) in the GDT group.

**Table 1:** Cost comparison in major reimbursement categories (in Euro)

	**GDT**		**Control**		
	***Mean ± SD***	***Median (IQR)***	***Mean ± SD***	***Median (IQR)***	***p value***
**Total**	**2877 ± 2336**	**2181 (1561–3154)**	**3331 ± 3238**	**2331 (1540–3687)**	**0.596**
Intraoperative*	**986 ± 351**	880 (744–1165)*	**830 ± 469**	688 (504–1006)*	**0.001**
Anaesthesia	493 ± 157	462 (389–591)	540 ± 184	495 (432–636)	0.094
Monitoring*	296 ± 0	296 (296–296)*	20 ± 0	20 (20–20)*	0.0001
Infusion	37 ± 17	45 (32–48)	32 ± 17	32 (19–48)	0.173
Blood products	160 ± 241	0 (0–255)	237 ± 336	80 (0–389)	0.264
**Postoperative**	**1891 ± 2170**	**1142 (764–2210)**	**2501 ± 3152**	**1626 (810–2367)**	**0.177**
**Patients care**	**505 ± 486**	**328 (218–582)**	**912 ± 1429**	**439 (222–921)**	**0.125**
Clinical examinations/procedures*	68 ± 177	29 (22–41)*	212 ± 593	38 (25–86)*	0.023
Biochemistry	205 ± 138	172 (117–246)	263 ± 271	174 (116–291)	0.702
Microbiology diagnostics and antimicrobials	99 ± 151	36 (17–136)	236 ± 535	62 (16 – 194)	0.365
Radiology examinations	34 ± 66	6 (6–30)	40 ± 68	6 (6–29)	0.944
Other	100 ± 122	38 (24–138)	161 ± 262	48 (26–190)	0.570
**Hospitalization costs**	**1386 ± 1736**	**831 (544–1570)**	**1589 ± 1863**	**1040 (491–1808)**	**0.459**
Intensive care unit stay costs	1173 ± 1736	637 (205–1480)	1240 ± 1752	704 (0–1409)	0.977
Ward stay costs	213 ± 108	202 (140–241)	349 ± 467	219 (161–368)	0.082

All p values are calculated using the Mann–Whitney test (* marks where p < 0.05).

Abbreviations: SD – standard deviation, IQR – interquartile range.

Additional costs induced by some specific complications or groups of complication were also evaluated. This analysis was limited by a low number of patients having one specific condition only. However, we were able to identify patients having just infectious complications (i.e. pneumonia, surgical site, urinary tract and catheter related blood stream infections), but only one non-infectious complication (i.e. acute heart failure). The occurrence of any complication, irrespective of study group allocation, increased the costs of postoperative care by 2295 ± 3611 € (p < 0.001) with a significant increase in all major cost driving categories. Mean costs for patients with and without complications are given in Table 2. For the groups with different complications the additional sum is also displayed to enable a direct comparison to data published by other authors [14,15].

**Table 2:** Postoperative costs among different complication subgroups (in Euro)

**Group**	**Total postoperative costs**	**Hospitalization costs**	**Patient care costs**	**Clinical examinations/ Procedures, etc.**	**Biochemistry**	**Antimicrobials ± microb. diagnostics**	**Radiology diagnostics**	**Other**
No complication N = 67	1182 ± 790	863 ± 656	320 ± 185	29 ± 14	152 ± 79	45 ± 54	15 ± 25	78 ± 98
Any complication N = 53	**3477 ± 3611**	**2278 ± 2387**	**1199 ± 1482**	**280 ± 640**	**337 ± 282**	**322 ± 561**	**64 ± 90**	**196 ± 277**
**Additional costs in specific groups**								
Infectious N = 31	**1789 ± 3262**	**1102 ± 2113**	**687 ± 1238**	**121 ± 304**	**123 ± 206**	**285 ± 660**	**36 ± 72**	**121 ± 239**
Non-infectious N = 7	**548 ± 657**	−48 ± 456	**597 ± 661**	**439 ± 641**	47 ± 154	**61 ± 112**	35 ± 63	14 ± 88
Multiple/mixed complications N = 15	**4155 ± 4434**	**2743 ± 2887**	**1411 ± 2065**	**433 ± 1028**	**375 ± 370**	**360 ± 446**	**81 ± 126**	**162 ± 391**
**Additional costs in specific complications**								
Pneumonia N = 6	1437 ± 4161	1118 ± 373	320 ± 695	26 ± 76	111 ± 244	**125 ± 202**	**11 ± 20**	46 ± 188
Surgical site infection N = 7	**2119 ± 1040**	**1486 ± 932**	**633 ± 274**	**84 ± 130**	**158 ± 98**	**192 ± 163**	**51 ± 80**	148 ± 183
CRBSI N = 9	643 ± 1314	399 ± 1138	**244 ± 276**	21 ± 40	42 ± 105	**132 ± 125**	22 ± 41	27 ± 155
UTI N = 9	175 ± 606	66 ± 400	108 ± 292	6 ± 11	75 ± 234	20 ± 111	2 ± 24	5 ± 84
Acute heart failure N = 4	**1152 ± 1182**	**843 ± 1017**	**309 ± 205**	**85 ± 113**	**139 ± 157**	**111 ± 88**	1 ± 10	−28 ± 48

Comparison between the No and Any complications group have been made using the Mann–Whitney test, all other comparisons between subgroups were assessed by the Kruskal-Wallis test using the Conover post-hoc analysis.

Bold type shows statistical significance against the patients without complications.

Negative number in additional costs: decrease in costs as compared to patients without complication.

Abbreviations: CRBSI – Catheter related blood stream infection, UTI – Urinary tract infection (including asymptomatic bacteriuria).

## Discussion

The current study evaluates in a retrospective fashion the economic implications of intraoperative fluid optimization guided by stroke volume variation based on previously published clinical results [12]. The mean cost per patient in the intervention arm was 2877 ± 2336 € compared to 3331 ± 3238 € in the control group. Overall, the incidence of postoperative complications was the most important cost driver. In addition, our results here detail the incremental costs due to different infectious and non-infectious complications which will enable a comparison of health care costs in post-communist European countries to those from the West.

Economic data and proof of economical benefit are lacking for many interventions from our daily practice. Nevertheless the current economic situation in most developed countries impacts budgets and so economic analyses of new and existing therapies are becoming increasingly important. This is perhaps more so in post-socialistic countries of Middle and Eastern Europe as the shortage of resources is most striking there. Perioperative goal directed hemodynamic optimization is one of the most promising concepts for clinical benefit in the postoperative period as its use has been shown to avoid complications, reduce patient length of stay and reduce postoperative morbidity. Alas, the concept is still not widely embraced and is seldom used outside specific health care facilities. Perceived increased economic burden, anaesthesiologist workload and a lack of proven economical benefit might be some of the reasons for not adopting this approach. It is true that there are only limited economic data to support perioperative GDT although the economic evaluations by Boyd [6,7] and Wilson [8,9] have both shown cost-effectiveness, but due to recent developments in the field they are of limited impact. Many different devices have since replaced the pulmonary artery catheter in routine use for GDT in high risk populations and the lower invasiveness increases the number of patients having a potential clinical benefit [16]. However, the acquisition costs of most of these devices and disposables used are deemed high and in many countries not reimbursed by the payer. The results of our study demonstrate that this is true to a degree and anaesthesia related costs are slightly, but significantly increased. This may be relevant when comparing patients without complications, but complication related costs highly exceed those of monitoring and GDT has been proven to reduce complications. This shows that even after inclusion of these costs the GDT approach would be cost-saving in populations with high postoperative morbidity.

Recently Bartha [11] published a large model based analysis showing that goal directed hemodynamic therapy would be a dominant strategy (i.e. both cost and life saving) in octogenarians undergoing hip replacement. Results from six meta-analyses and 17 research papers (using different approaches and devices) were taken as model inputs and an overall reduction of medical care costs by 1882 € was demonstrated. In 2007 a health technology assessment of the oesophageal Doppler was published by Mowatt [10]. As no formal economic data were available the authors constructed a model based on eight randomized single centre studies. The GDT approach was found likely to be cost-effective even for the worst model scenario. Since then NICE has positively appraised this, but also expanded it to include other devices (including Vigileo/FloTrac). Our patient-specific “real world” data validate these model based assumptions, even though the exact monetary sums differ. Health care costs in Sweden were taken as economic inputs in the former and ward/ICU stay hospital costs in United Kingdom in the later study. Our real, clinically derived economic data from the Czech Republic seem to be closer to Mowatt’s worst scenario but do not take into account purchasing power or other translations of economic value.

When trying to interpret the results from our study it is important to consider some specifics of the health care system in the Czech Republic. For example, it’s likely that the honoraria and costs of human work are lower in former Eastern bloc European countries than other Western systems. However, the costs of materials and drugs used are potentially more similar to those in the West. This is important to understand when considering those activities in where human work creates the major part of care (e.g. clinical examinations, laboratory or radiology assessments). However, the extent to which any management practices are employed as standard of care are also pivotal to interpreting economic data in critical care. As a direct comparison of complications costs are unavailable, we tried to calculate the additional costs of different complications to enable this East-to-West comparison. An Italian study concerning perioperative nutritional support in gastrointestinal surgery (Braga et al. [14]) and a French analysis of nosocomial infections costs [15] served as comparators. The first study showed slightly shorter length of stay after colorectal surgery compared to our GDT surgical group (8.8 days in Braga vs. 9.9 days), but the average costs were far higher in patients with complications (10,494 € vs. 5,419 €) and to a lesser extent also in those without complications (2,552 € in Braga compared to 1,956 € in our study population). The costs of different individual complications were 2–10 times higher than those observed in our patients. The French study [15] allows a better differentiation between major cost drivers pointing out the stated difference in costs of resources and clinical examinations/procedures. Alas, costs of hospitalization (the major cost-driver in patients with infection in our population) are not indicated separately. The reported additional charges were comparable in terms of antimicrobial agent costs but conversely the clinical, laboratory and radiology assessment expenditures were 2–10 times lower in our evaluation. According to these results, higher cost-savings (resembling the data published by Bartha [11] and Mowatt [10]) would have been calculated in the same population when using Western European countries complications related rates.

Some other factors and assumptions made in our analysis have to be commented in order to address potential bias. We have excluded the costs of the preoperative hospitalization from our analysis. The hemodynamic intervention cannot affect the preoperative course and hence we deemed these costs to be source of potential bias in the assessment due to the relatively small sample size and potential variability in cost. Similarly the costs incurred by the primary surgery itself were excluded from the analysis. Almost two thirds (79 patients, 66%) of our patients underwent intra-abdominal vascular surgery. The costs of the material used (grafts and sutures) highly offset the other costs (i.e. anaesthesia, monitoring). Their inclusion may have had a severe impact on the final sum without being relevant to the subject under study. In contrast, the costs of any further surgery related to potential complications were included in the analysis. Another potential limitation is the additional medication given to the patient. This is mostly charged as a lump sum per hospitalization day. Only specific treatment groups (i.e. antimicrobial agents, blood products, chemotherapeutics) are directly reimbursed by the insurance companies. When the treatment cost exceeds the *per diem* sum the deficit is covered by the hospital budget. The knowledge of the exact sum spent on the medication would be important for the hospital perspective but we were unable to extract the data necessary for this calculation. This was one of the major reasons for performing the healthcare payer’s perspective analysis, which remains unaffected by the missing information.

Retrospective evaluation of economic data poses another potential flaw. We have managed to collect all the relevant information in 116 (97%) patients. In four patients (three from the Control group and one from the GDT group) who were transferred into another healthcare facility, only the data coming from the original hospital were available. Social reasons played an important part in two of these hospitalisations (one case in each group) and potentially irrelevant but in the remaining two cases (both from the Control group) the patients spent 12 and 15 days in tertiary hospitals to treat some resulting complications. We consider this the conservative approach as it clearly biases against GDT and although costs related to these hospitalizations were not available for assessment, the missing economic data could only add to the observed benefit.

With regards to other limitations of our study, firstly, the data are based on the costs directly charged to the healthcare payer. Other costs to the hospital ward and staff, patients and other possible subjects were not included as well as the long term impact of potential decreased productivity and other indirect costs. Second, the analysis was based on the results of a single centre study performed in the Czech Republic and although this undoubtedly leads to better control of the study, transferability of results is not automatically relevant to other centres. Finally, although the point averages and trends for all results were consistent in their direction (i.e. favouring GDT) only the difference in the postoperative clinical examinations and procedures reached statistical significance. In the original study design the economic endpoints were not included in the statistical power calculation and according to our observations a study including more than 200 patients in each arm would be necessary in order to reach a statistical significance in major economic endpoints.

Nonetheless, in the absence of prospectively collected economic data from other clinical studies we present here what we consider to be some of the best evidence available on which to assist clinical decision making where economic constraints are present. We have actively attempted to remove any potential bias and in order to produce a conservative evaluation have selected the options that favour the Control group when faced with a choice.

On balance, the implications for clinical practice based on the results of this economic analysis alongside the clinical data are strongly in favour of GDT. Where the lack of proven economic benefit and need for initial investment are limitations for successful implementation of perioperative GDT we have shown that a conservative estimate produces a strong trend in favour of GDT. Costs associated with GDT implementation were offset by reduced costs of postoperative care and this may help to convince hospital administrators and healthcare payers that implementing perioperative GDT is not only beneficial for patients’ health but also economically attractive.

## Conclusion

Intraoperative fluid optimization with the use of stroke volume variation and Vigileo/FloTrac system showed not only a substantial improvement in morbidity, but was associated with an economic benefit. The mean savings observed in the overall costs of postoperative care (namely clinical care, costs of antimicrobial treatments and ward stay costs) trend to offset the investment needed to perform the GDT strategy and intraoperative monitoring. In addition, important differences in complication associated costs between East and West European countries were observed.

### Key messages

• Perioperative goal directed therapy was associated not only with clinical, but also important economic benefit.

• The cost-savings observed in the overall costs of postoperative care (namely clinical care, costs of antimicrobial treatments and ward stay costs) trend to offset the investment needed to perform the goal directed therapy strategy and intraoperative monitoring.

## Abbreviations

CI: Cardiac index; CRBSI: Catheter related blood stream infection; UTI: Urinary tract infection; CZK: Czech crowns; €: Euro; GDT: Goal directed therapy; ICU: Intensive care unit; NICE: National institute for health and clinical excellence; SD: Standard deviation; SVV: Stroke volume variation; SVVOPT: Acronym of the “Intraoperative fluid optimization using stroke volume variation in high risk surgical patients” trial; QALY: Quality adjusted life years.

## Competing interests

JB Received lecturing fees and is member of the advisory board of the Edwards Lifesciences, all other authors declare NO competing interests.

## Authors’ contributions

JB designed the study, performed the calculations and drafted the manuscript; JZ and AS performed the data extraction and analysis; ICh and EK participated on the study design, helped to analyse the data and write the manuscript. All authors read and approved the final manuscript.

## Pre-publication history

The pre-publication history for this paper can be accessed here:

http://www.biomedcentral.com/1471-2253/14/40/prepub
